# Feasibility of Using 3D Printed Polyvinyl Alcohol (PVA) for Creating Self-Healing Vascular Tunnels in Cement System

**DOI:** 10.3390/ma12233872

**Published:** 2019-11-23

**Authors:** Zijing Li, Lívia Ribeiro de Souza, Chrysoula Litina, Athina E. Markaki, Abir Al-Tabbaa

**Affiliations:** Department of Engineering, University of Cambridge, Trumpington Road, Cambridge CB2 1PZ, UK; lrds2@cam.ac.uk (L.R.d.S.); cl519@cam.ac.uk (C.L.); am253@cam.ac.uk (A.E.M.); aa22@cam.ac.uk (A.A.-T.)

**Keywords:** polyvinyl alcohol (PVA), vascular networks, 3D printing, self-healing, cementitious materials

## Abstract

Pursuing long-term self-healing infrastructures has gained popularity in the construction field. Vascular networks have the potential to achieve long-term self-healing in cementitious infrastructures. To avoid further monitoring of non-cementitious tubes, sacrificial material can be used as a way of creating hollow channels. In this research, we report a new method for fabrication of complex 3D internal hollow tunnels using 3D printing of polyvinyl alcohol (PVA). The behaviour of 3D printed PVA structures in cement pastes was investigated using computed-tomography (CT) combined with X-ray diffraction (XRD), Fourier-transform infrared spectroscopy (FTIR), and scanning electron microscopy with energy dispersive spectroscopy (SEM/EDX). Results showed that (i) 1300 min were needed to fully dissolve 1 g of a 3D printed PVA structure, and different pH solutions did not significantly change the PVA dissolving process compared with a neutral environment; (ii) a low water/cement ratio can minimize early stage cracking resulting from PVA expansion; (iii) and PVA-cement interaction products were mainly calcite and a Ca-polymer compound. In conclusion, controlling the PVA expansion by decreasing the water/cement (w/c) ratio provides a promising approach to achieve 3D hollow channels in cement and, therefore, makes it possible to create complex tunnels within self-healing cementitious materials.

## 1. Introduction

Most of our infrastructure, including skyscrapers, mega dams, and super highways, is made out of cement-based materials (stabilized soil, soil cement systems, grouts, and concrete). However, cement-based infrastructure deteriorates over time under an aggressive condition [1]. The main concern in building construction is crack initiation by thermal effects, early-age shrinkage, and mechanical loading [2]. In the European Union 20% of all concrete repair works fail in the first five years, 55% fail within the first 10 years, and all fail within 25 years. This leads to significant maintenance and repairing costs. Maintenance costs of existing cement-based structures accounts for approximately 45% of the total expenditure of the construction industry, at around 40 billion pounds per year, in the UK [3]. Traditional repairing works mostly requires workforce to reach the damage place, which makes it almost impossible to access infrastructures such as a radioactive disposal container, underground space, and tunnels. Recent advances in smart materials have led to the emergence of the field of self-healing materials and biomimetic structures in general.

The healing process in cementitious material is divided into two categories: autogenous healing, which is related to intrinsic properties of cementitious materials, and autonomic healing, which is associated with embedding healing agents in the matrix [4]. However, autogenous healing is limited by crack width. The crack needs to be roughly below 200 μm [5]. As for autonomous healing, an encapsulation technique was one of the successful cases based on the release of limited quantities of a stored healing agent from polymer capsules [6,7,8], glass capsules [9,10], ceramic capsules [11], and mineral capsules [12]. However, this technique has difficulties in ensuring long-term service life and repeated damage repair due to its uncertainty of capsules breaking and limitation in providing enough healing agents for large cracks.

In this case, the vascular system is the one to overcome those difficulties. Vascular systems normally have interconnected networks that are incorporated within the cement matrix. This structure makes it possible to diagnose cracks more effectively when compared with discrete capsules distributed in the matrix. With a circulation system, healing agents could be recycled and refined, which provide a way for continuous healing agent delivery.

There are two main ways to create vascular structures in cementitious materials: remaining glass/plastic tubes in-situ and removing the none-cementitious tubes after pastes are cured. Early research studies such as the one from Dry et al. [13,14] mainly focused on the first in-situ approach. Following this approach, researchers such as Joseph [15] further developed one-dimensional (1D) glass tubes with outputs opening to the air. 

For the second method, it avoids long-term tube monitoring and enables multi-scale healing over time. Studies [16] currently apply polyurethane (PU) tubes and pull them out after cementitious pastes are cured since PU tubes will shrink after heating. This removable 2D PU structure provided a way of maintaining multi-use networks that can be re-used over the lifetime of a cementitious structure to enhance and enable multi-scale healing. However, this is only for one-dimensional and two-dimensional (1 and 2D) vascular structures.

On the other hand, three-dimensional (3D) networks are more complicated and consist of interconnected structures, which are capable of transporting the healing agent to the damaged areas across the specimen’s volume. However, it is also hard for 3D vascular structures to monitor glass/plastic tubes after several healing cycles. This means we need to create hollow internal tunnels in a three-dimensional way. Removable PU tubes can only be used in simple structures like single channels or 2D grid structures. This is because they cannot be bent to a high curvature to create complex or biomimetic structures. Such complicated structures would make it even harder for pulling PU tubes out after pastes are cured. 

In this study, polyvinyl alcohol (PVA) feasibility as a sacrificial material to create 3D internal tunnels in cementitious system was investigated. PVA is a widely used medical application, such as eye drops, with biocompatibility and low toxicity [17]. Thus, it was chosen as an ideal sacrificial material for creating hollow channels with low healthy risk to workers and concrete casters. The advantages of such an approach are: (i) it uses commercial 3D printing material and can print complex 3D structures; (ii) PVA is water soluble and, therefore, can be easily removed from prisms using water. A PVA double twisted channel was designed and 3D-printed, due to its potential for delivering two-part healing agents. The crystallinity and functional groups of PVA before and after extrusion were investigated using FTIR. The dissolution of PVA structures in water was also demonstrated. The behaviour of the 3D-printed structures during the curing of cement paste at different water content was investigated using optical microscopy and computed-tomography (CT). PVA reacted with cement paste during the 28 days of curing and the reaction products were analysed using SEM-EDX, FTIR, and XRD. The research focuses on the potential of PVA to be used as a sacrificial material to create hollow tunnels in the cementitious material to promote self-healing. 

## 2. Materials and Methods

### 2.1. Fabrication of 3D Printed PVA Vascular Structures and Cement Samples

**Figure 1:**
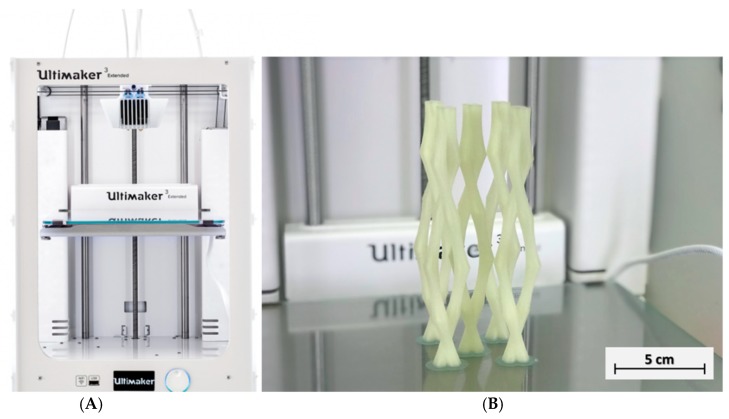
(**A**) 3D printer and (**B**) double twisted channel 3D-printed PVA model.

The vascular networks were designed in AutoCAD^®^ and then printed using an Ultimaker^®^ 3 Extended 3D printer (Fused deposition modelling system, FDM, Ultimaker, Utrecht, Netherlands) and Cura^®^ software. Polyvinyl alcohol (PVA, by Ultimaker^®^, Utrecht, Netherlands) filaments were used to produce vascular templates. During the printing process, the PVA flows through a heated nozzle (0.4 mm nozzle, 215 °C) and deposits a layer (0.1 mm thickness) of constant thickness in the x-y plane. Once a layer has been deposited, the print plate moves down a distance equal to the material layer thickness and the next layer is deposited, at a printing speed of 70 mm/s. Figure 1 shows the three-dimensional vascular network built up with a double twisted channel designed for delivering a two-part healing agent. The PVA diameter was chosen as 4 mm based on previous results reporting that a 2-mm tube diameter is necessary for releasing the healing agents from the tube [18].

### 2.2. The Cementitious Matrix

**Figure 2:**
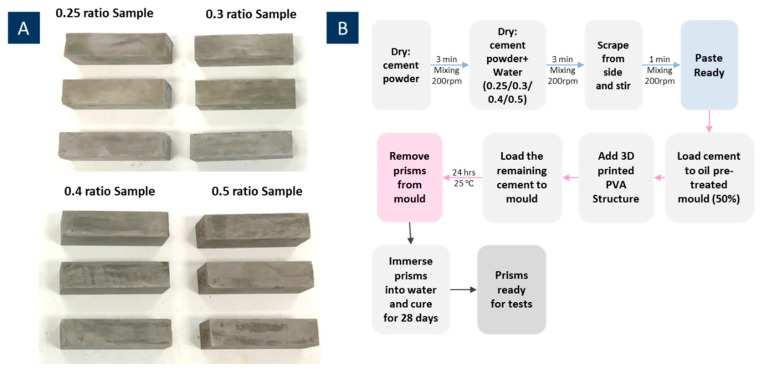
Photography of produced PVA prisms (**A**) and fabricated procedural figure (**B**).

Prismatic specimens were prepared for testing the viability and performance of the 3D printed polyvinyl alcohol (PVA) vasculature within a cement matrix. The cement paste mix was prepared with Ordinary Portland Cement (PC) (CEM-I, 52.5, Hanson, Parramatta, Australia) and water at water/cement (w/c) ratios of 0.25, 0.3, 0.4, and 0.5 since the flow of the cementitious mix will play an important role in the appropriate formation of the cement around the vascular structure. Stainless-steel moulds (40 mm × 40 mm × 160 mm) were used for preparing the prismatic cementitious beams. First, prism moulds were cleaned and slightly lubricated with de-moulding oil. PC cement in dry conditions was stirred for 3 min. Then water was added using a planetary type mixer at 200 rpm. Following the mixing, moulds were 50% filled with the cement paste before adding 3D printed PVA structures. Then the remaining parts of cement pastes were loaded into the moulds with PVA structures settled (Figure 2).

### 2.3. Characterization Methods 

Crack formation was observed using a DM 2700 M Leica^®^ microscope (Wetzlar, Germany) equipped with LAS v4.6 software. Images of cracks during the curing process were captured and monitored over time at 1 day, 2 days, 5 days, 10 days, and 28 days. In the microstructure of the hydrated cement, mineral and self-healing materials were investigated using XRD, FTIR, TGA, and SEM-EDX analyses. For XRD, samples were mounted on a smooth glass surface attached to a flat holder and examined using a Siemens D500 X-ray diffractometer (Munich, Germany) with a Cu-Kα radiation source. The diffractometer was operating at 40 kV and 40 mA, and emitting radiation at a wavelength of 1.5405 Å. Scanning ranged between 10–60°(2θ) at a rate of 1 s/step and a scanning resolution of 0.02°/step. Following the scanning, the diffraction raw data were searched in the PDF-2004 database using the Jade^®^ software in order to identify the material peaks in the graph.

Infrared spectroscopy was used to further characterise the PVA structure in terms of fundamental vibrations and the associated behavior of the rotational-vibrational structure. A mid-infrared (4000–400 cm^−1^) ranged Perkin Elmer FTIR Spectrometer Spectrum Two (MA, USA) was used to perform this test. Spectra were collected in the transmittance bands between 4000 and 400 cm^−1^ at a resolution of 1 cm^−1^. SEM-EDX was carried out using Phenom (Pro G2, Eindhoven, The Netherlands). An electron beam energy of 15 keV with a short working distance (~6–8 mm) was used for all imaging. Samples were coated with gold film (2–3 nm) using a K550 Emitech sputter coater to avoid charging and improve the secondary electron signal required for topographic examination in the SEM. The cement samples were scanned at Nikon on an X-Tek H225 ST CT-scanner (Tokyo, Japan). Reconstruction of the original Computed tomography (CT) images produced 1300 slices (oriented obliquely through the specimen) composed of isometric voxels with a resolution of 0.089579 mm/voxel. CT scans were processed using the 3D visualization software package Mimics v.14.0 (Materialise HQ, Leuven, Belgium) on a 64-bit Dell Inspiron 15R laptop with 8 GB RAM.

## 3. Results and Discussion 

### 3.1. Chemical Property and Dissolution Behaviour of the 3D Printed PVA

A non-disclaimed PVA compound filament was investigated in order to extrude it in a commercial 3D-printer. PVA is widely used in the 3D printing field as a supporting and break-out material due to its brittleness and ability to dissolve in water [19,20]. However, it is less popular and has limitations in terms of a different nozzle requirement compared to other traditional 3D printing materials such as polylactic acid (PLA) and acrylonitrile butadiene styrene (ABS). Heating the PVA in the same PLA/ABS extruding print resulted in bubble formation and clogs in the extruder [21]. Thus, the chemical properties of the non-disclaimed PVA provided before and after the extrusion were first compared and then the dissolution behaviour of the former was investigated.

**Figure 3:**
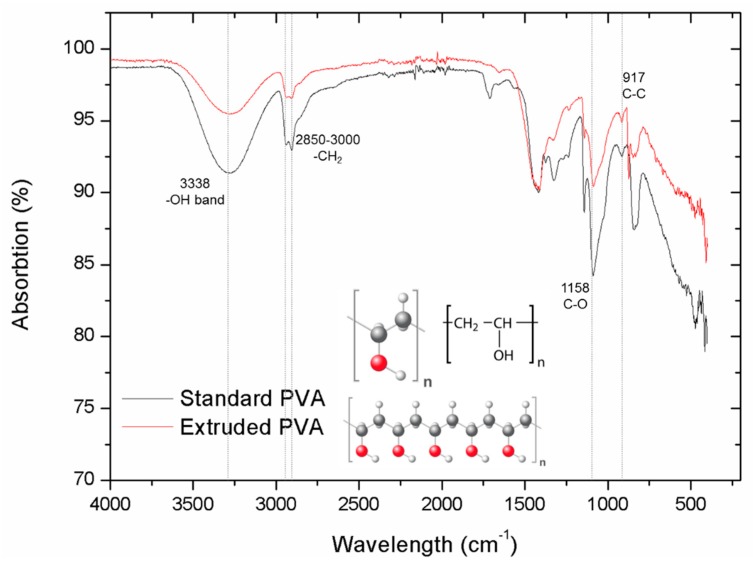
The FT-IR spectra of the 3D extruded and standard PVA materials.

The chemical functional groups in PVA before and after 220 °C extruding were compared using FTIR (Figure 3). The sharp peaks at around 2850 and 3000 cm^−1^ are assigned to the CH_2_ stretching modes [22,23]. In addition, a hydroxyl group contribution was seen with absorption at around 3338 cm^−1^. Important peaks at around 1158 were verified as the C–O bond. C–C bonds with a frequency at around 917 cm^−1^ were identified previously in FTIR data [24]. Extruded PVA showed different peak intensities compared to the original PVA whereas the peak wavelengths remained the same. Peak intensity differences were mainly affected by the crystalline portion of the polymeric chains. Additionally, the peak at 3338 cm^−1^ broadened and indicated that the intramolecular hydrogen bond is formed between two neighbouring OH groups [23]. This means that the extrusion process involved in 3D printing slightly changed its crystallinity. Furthermore, the main functional groups remained unchanged. In this case, there was no significant difference between standard PVA and extruded PVA, so that standard PVA behaviours are also applicable for the extruded PVA.

**Figure 4:**
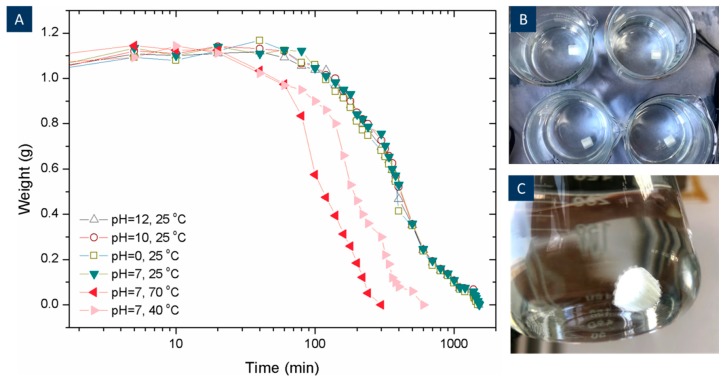
3D printed PVA cylinders dissolving behaviour in different temperatures and pH (**A**), photograph of 3D printed PVA cylinder dissolving in different pH solutions (**B**), photograph of 15 mins of dissolving when the expansion layer appears (**C**).

The dissolution of PVA was investigated by 3D printing PVA cylinders (1 mm in length and 1 mm in diameter, 1 g) and measuring it weight variation while submersed in water, as shown in Figure 4. The samples were tested at 25 °C at a pH of 0, 7, 10, and 12 and also tested at increased temperatures of 40 °C and 70 °C for samples at a pH of 7. The weight of each cylinder was measured every 5 min for the first 30 min, and every 15 min until it was fully dissolved [25]. The results are presented in Figure 4. The results show that all the 3D printed PVA cylinders showed an increase in weight at the early stage of the test before the weight started to reduce. This is due to water uptake by the PVA, which enables water molecules to penetrate its polymeric chains. Therefore, this weakens the –OH bonds and then dissolves PVA [26]. It is clear from the image that the pH does not have an effect on PVA dissolution or timescales. This showed that the highly alkaline environment of the cement paste or any changes in the pH would not affect the dissolution of the PVA, and the main PVA structures can survive in the cement paste during the curing process. The temperature, on the other hand, was found to accelerate the dissolution process. The PVA structures were dissolved within 5 h at 70 °C, while it took 25 h at room temperature to dissolve the structures.

**Figure 5:**
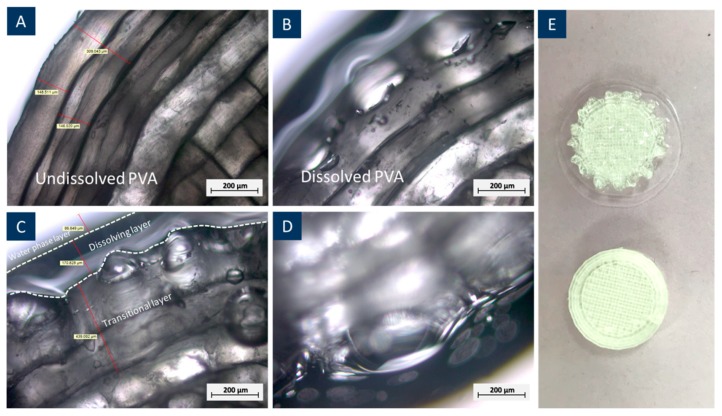
3D printed PVA cylinders dissolving behaviour under an optical microscope. (**A**) Undissolved PVA, (**B**) dissolved PVA when weight reached 20% of its original weight, (**C**) layer classification of PVA dissolving, (**D**) detailed image of the dissolving layer, (**E**) general image of dissolved (top) and undissolved PVA cylinders.

The water absorption of PVA when immersed in water leads to the volume expansion of the 3D printed structures. This was found in the early stage of the PVA dissolving test, where the weight of PVA structures peaked at around 1.2 g, at nearly 20% of its initial weight. Figure 5 shows microscopic observations of PVA before and after dissolution, confirming the expansion as the outer layer of PVA formed a transitional zone of a semi-liquid phase, where –OH took up water molecules but did not yet transform into a liquid phase [27]. This is the main reason of volume expansion at the early stage of dissolution.

### 3.2. PVA Expansion in Cement Prisms

**Figure 6:**
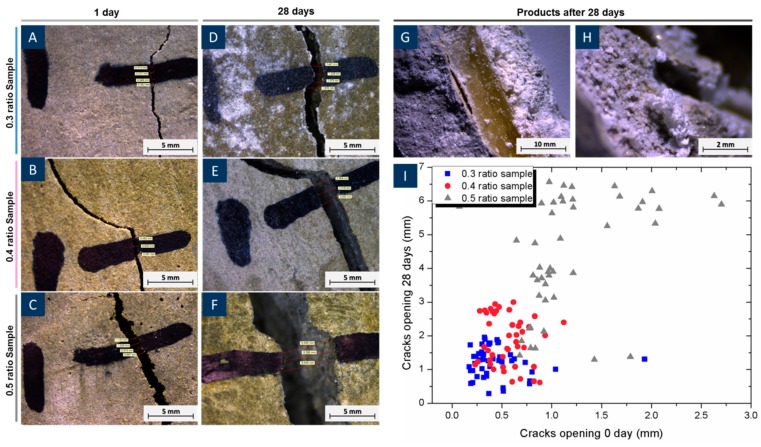
Surface crack widths of specimens with different water/cement ratios under the microscope after 1 day of curing (**A**–**C**), 28 days of curing (**D**–**F**); Photography of reacted products (**G**,**H**) and Crack width diagram (**I**).

To examine the behaviour of PVA structures during curing, the 3D printed structures were embedded in cement paste with four different types of water/cement ratios, which are 0.25, 0.3, 0.4 and 0.5, in accordance with BS EN 196-1:2005 [28]. Micro-surface cracks were found after samples were demoulded with water/cement ratios of 0.3, 0.4, and 0.5 (Figure 6). However, no cracks were observed from 0.25 samples. The majority of the initial surface crack widths were within 0.7 mm for samples with water/cement ratios of 0.3 or 0.4. As for samples with a water/cement ratio of 0.5, widths of initial surface cracks were mostly between 1 to 2 mm. After 28 days of curing, with the specimens immersed in water, cracks expanded dramatically in the samples with water/cement ratios of 0.4 and 0.5. However, surface crack expansion in 0.3 water/cement ratio samples was controlled within 2 mm. This is because low water/cement ratio samples were dense and can somehow restrict the PVA expansion. Additionally, they had a large amount of unhydrated cement compared with that in high water/cement ratio samples, so that more water was used for the hydration process. Light-coloured secondary products were found by the edge of PVA and cement under the microscope. This part will be discussed in detail in the next section. These indicated that a high water/cement ratio would result in larger early stage cracks due to a high amount of water in cement pastes provided for PVA expansion. However, early age cracks can be limited by applying a low water/cement ratio, and can even be avoided when a 0.25 w/c ratio was utilized.

**Figure 7:**
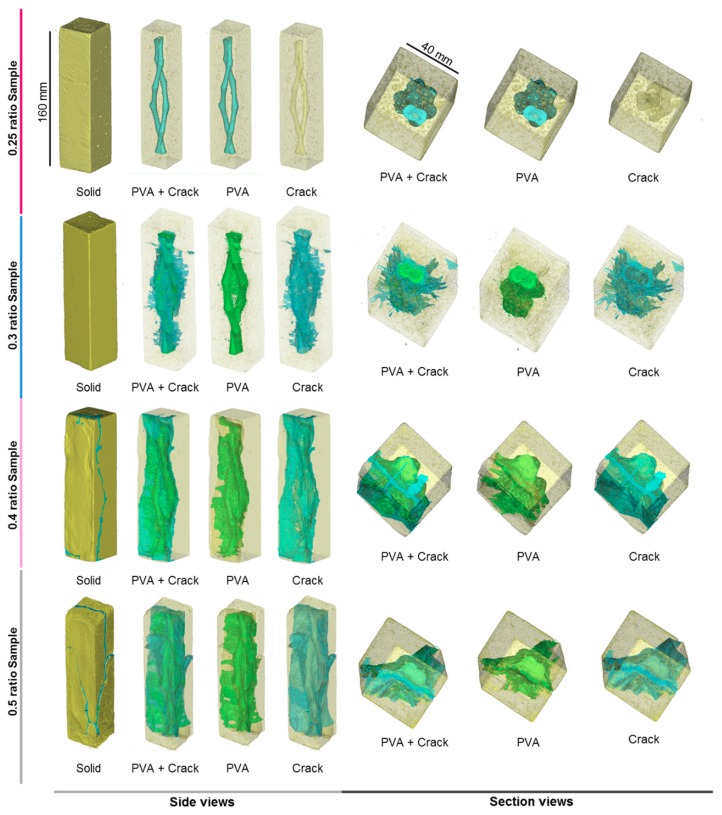
3D reconstructed images of reacted cement samples (turquoise represents the PVA structure in a 0.25 sample, yellow bulk shows cement prisms are shown in yellow bulk (all samples), the PVA structures in Figure 1 are shown in green (sample 0.3, 0.4 and 0.5), the cracks are shown in light blue (sample 0.3, 0.4, and 0.5; 0.25 has no cracks).

To investigate the effect of 3D printed PVA structures in cement, we used computed tomography (CT) for the four types of w/c ratio samples. From the CT scanning reconstructed images (Figure 7), three different materials could be identified in terms of density difference: yellow bulk represented cement paste, PVA related material were shown in green, and light blue reflected cracks and dissolved empty space in cement paste, with the bubbles removed from the crack identification. In the 0.25 sample, the PVA structure was represented in turquoise, as there were no cracks appearing within the cement paste. 

Microcracks in samples with cement ratios of 0.3, 0.4, and 0.5 were formed and increased in length during the 28 days of curing process, as observed before with optical microscopy. Early PVA expansion resulted in free space. This was because PVA absorbed water from the cement mixture and then expanded their volume by taking up more space. During this process, cement got harder and kept its shape, even though the volume of PVA shrank due to a dissolved part of PVA travelled out of pastes through cracks. However, the general shape of the PVA structure in 0.25 cement paste remained mainly unchanged compared with the one in 0.3, 0.4, and 0.5 cement pastes. In addition, there were no significant cracks identified in the sample with a water/cement ratio of 0.25. This suggests that low water/cement ratio pastes could significantly minimize and even avoid early stage cracking during the curing process and retain the original shape of the PVA structures.

### 3.3. Interaction between PVA and Cement

**Figure 8:**
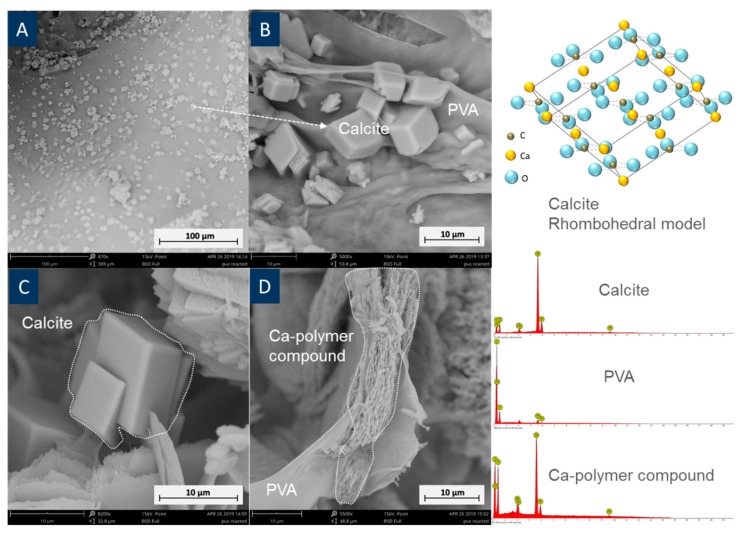
SEM images of cement samples reacted with 3D printed PVA structures showing (**A**) boomed calcite minerals with a PVA background, (**B**) zoomed in the image of PVA and calcite, (**C**) calcite crystals, and (**D**) Ca-polymer compound generated on PVA. A calcite crystal rhombohedral model and EDX spectra corresponding to calcite, PVA, and a Ca-polymer compound are also shown.

After samples were cured for 28 days, products from PVA and cement under the microscope were analysed by SEM-EDX, FTIR, and XRD. This was done to further understand the chemical components and 3D PVA-cement interaction mechanism. EDS showed that calcite and Ca-polymer compounds were found in all specimens, as illustrated in Figure 8. 

**Figure 9:**
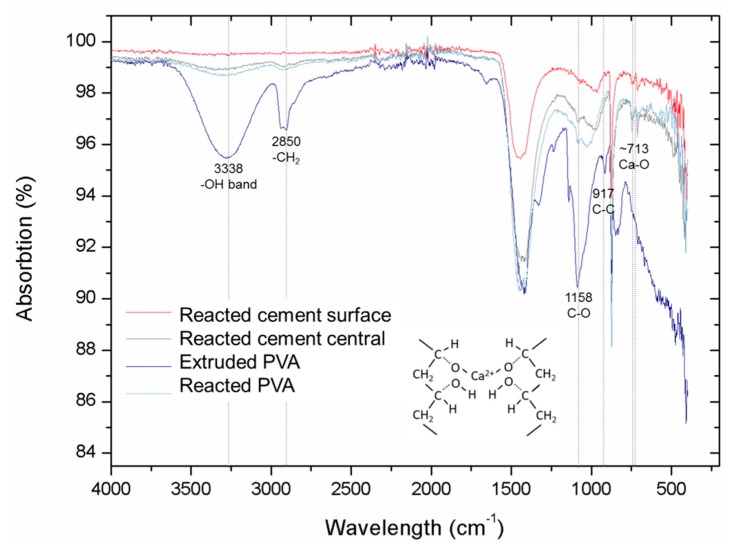
FT-IR spectra of cement samples reacted with 3D printed PVA structures.

The existence of the Ca-polymer compound was also confirmed by FTIR (Figure 9). The peak at around 713 cm^−1^ corresponds to the Ca–O bond [29] and this can be found in a reactive PVA sample. This strongly suggests that there is an interaction between PVA and the Ca cations. Both the surface and the central cement parts reacted with PVA, since minor Ca–C bonds can be found in both FT-IR curves. The surface cement reacted with the surrounding water, which contained PVA polymers. Hydroxyl groups in reacted PVA were barely verified in FTIR compared with that from both standard PVA and 3D printed PVA samples. This is because –OH groups were replaced by Ca cations, forming the Ca-polymer compound or were lost and then formed water.

**Figure 10:**
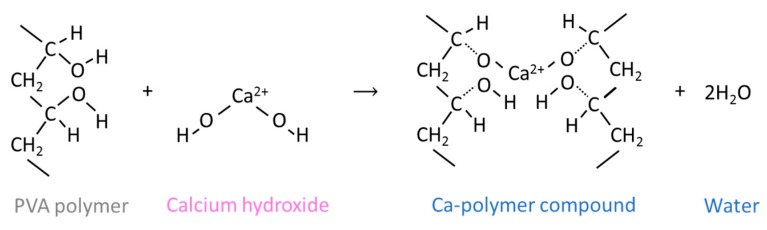
Reaction between PVA polymer and Calcium hydroxide, modified from Bonapasta et al. [27].

Bonapasta et al. found the similar secondary products in PVA cement interaction. This reaction process can be explained by Figure 10, where Ca^2+^ from calcium hydroxide replaced H^+^ from the PVA polymer, the replaced H^+^, and the remaining OH^–^, which then resulted in water [27]. 

**Figure 11:**
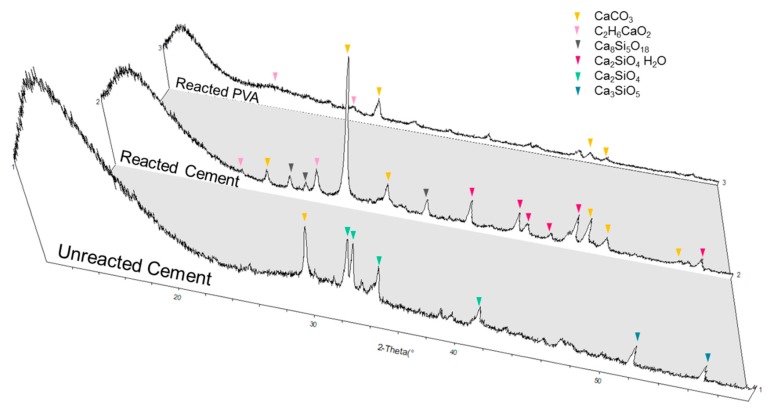
XRD diagrams of cement samples reacted with 3D printed PVA structures.

This was supported by XRD analysis (Figure 11). Ca-compound (Calcium methylate, C_2_H_6_CaO_2_, PDF card 19-1573) was identified using Jade^®^ software (ICSD database), in a reacted cement sample, while these were not observed in the unreacted cement. Calcium methylate could be one of the forms of the Ca-compound (R-(CHO)_2_-Ca), due to varied chain lengths [17]. 

Formation of this secondary material suggests that PVA lost H and hydroxyl groups in forming the Ca-compound and water. This coincided with the FTIR data (Figure 9), which showed a drastic decline of –OH group intensity between unreacted and reacted PVA. A significant increase of calcite in the PVA-cement reacted sample was found as compared to a plain cement sample. Additionally, calcite peaks were found in PVA polymer after a reaction with the cement, which was not present previously. This implies that the reaction between PVA polymer and Ca(OH)_2_ somehow concentrated Ca cations near the PVA polymer. When CO_2_ dissolved in water, it can easily react with Ca cations near PVA polymers.

### 3.4. Comparison with Existing Techniques and Cost Anaylsis

The specific feature for a self-healing vascular channel allows cementitious materials to sustain their properties from damages and avoid long-term tube monitoring. Compared with existing techniques in creating channels in cementitious materials, using PVA as sacrificial material makes it possible to create 3D channels and enable shape flexibility. The technique [16] relying on pulling out Polyurethane (PU) tubes by shrinking them after heating has more restrictions in terms of the shape design and tube dimensions. However, using PVA for creating channels also has a limit in the water/cement ratio choosing. 

As for the price in the main PVA vascular structures, current price is around 30–60 pounds per kilogram. This is much higher compared with the price of PU tubes, which only need around $2.95/ft USD. However, with the increasing popularity of 3D printing technology, the price of the PVA vascular channel is expected to decline over time. Additionally, the demand of the concrete repairing market is impressive. M&M (Markets and Markets) [30] reports that market size of concrete repair is estimated to grow from $1.69 Billion USD in 2015 to $2.62 Billion USD by 2021, at a CAGR (Compound annual growth rate) of 7.67%, in terms of value. The market is driven by increased spending on repair and maintenance of building and construction and rising trend of public–private partnership in the transport infrastructure in a developed country. Presumably, the traditional concrete repair market would be gradually replaced by self-healing technology, so that a continuous self-healing structure will be an important and distinctive aspect in the near future.

## 4. Conclusions

Creating hollow channels for a self-healing vascular system avoids long-term tube monitoring and enables multi-scale healing over time. The present study has indicated our initial effort of using PVA as complex tunnel creating material. PVA was selected as sacrificial material since it allows the 3D printing and the dissolution in water to create hollow tunnels. FTIR shows decreases in crystallinity of the PVA extruded in the 3D-printing, when compared with un-extruded PVA, but the main functional groups remained unchanged. We design and 3D-print a PVA double twisted channel since it provides a versatile way to deliver two-part healing agents. The resulting PVA structures completely dissolve in water. For example, 1 g 3D printed PVA structure dissolves in water after 1300 min. Water uptake followed by dissolution can be used as a simple removal mechanism for these vascular networks. Additionally, the highly alkaline environment of the cement paste does not affect the dissolution of the PVA. We embed the PVA structures in cement paste to investigate the effect of structures’ water uptake during curing. Through casting in different w/c ratios, we show the w/c ratio substantially contributes to increasing the water intake of the PVA structure. Controlling the PVA expansion by decreasing the w/c ratio provides a promising approach to tailor dissolution kinetics during curing. Furthermore, CT-scan images show that, with low w/c ratio casting, no dissolution of PVA is observed in the sample. On the other hand, for a w/c ratio above 0.3, the water uptake of PVA results in the expansion and creates cracks in the whole structure. Lastly, interactions between cement, mainly Ca(OH)_2_, and PVA mostly formed a Ca-polymer compound and water. Calcite was also found in reacted PVA polymers and reacted cement pastes due to air exposure. 

The capability of PVA to be 3D-printed, dissolved in water, and remain intact when casted at low w/c ratios should make it valuable as sacrificial networks for vascular-based self-healing. However, methods for preventing early stage cracking have not been studied in this research. Future studies include the effect of the hollow vascular networks on the mechanical properties and the pumping of the healing agent through the vascular network to evaluate self-healing performance.
